# OMERACT: An international initiative to improve outcome measurement in rheumatology

**DOI:** 10.1186/1745-6215-8-38

**Published:** 2007-11-26

**Authors:** Peter Tugwell, Maarten Boers, Peter Brooks, Lee Simon, Vibeke Strand, Leanne Idzerda

**Affiliations:** 1 Department of Medicine, University of Ottawa and Institute of Population Health, Ottawa, Ontario, Canada; 2 Department of Clinical Epidemiology and Biostatistics, VU University Medical Centre, Amsterdam, The Netherlands; 3 Faculty of Health Sciences, The University of Queensland, Brisbane, Queensland, Australia; 4 Harvard Medical School and Beth Israel Deaconess Medical Centre, Boston, MA, USA; 5 Division of Immunology, Stanford University, CA, USA; 6 Institute of Population Health, University of Ottawa, Ottawa, Ontario, Canada

## Abstract

OMERACT is the acronym for an international, informally organized network initiated in 1992 aimed at improving outcome measurement in rheumatology. Chaired by an executive committee, it organizes consensus conferences in a 2-yearly cycle that circles the globe. Data driven recommendations are prepared and updated by expert working groups. Recommendations include core sets of measures for most of the major rheumatologic conditions. Since 2002 patients have been actively engaged in the process.

## What is OMERACT?

"Clinical trials are only as credible as their endpoints" [1]. OMERACT strives to improve endpoint outcome measurement through a data driven, iterative alignment process as described below. The key characteristics of OMERACT include a commitment to the data-driven interactive development of a majority alignment across relevant stakeholder groups on determining relevant health outcome domains and endorsing valid, responsive, feasible health outcome measures/scales in patients with musculoskeletal conditions.

The first conference on rheumatoid arthritis was held in Maastricht, the Netherlands in 1992. The motivation for this were discussions between two of the authors, [MB and PT], comparing the outcomes used in European clinical trials of rheumatoid arthritis with that of North American clinical trials, and noting that they used different endpoints. This made it extremely difficult to compare and combine in meta-analyses. One of the authors had had a good experience with the nominal group technique to build consensus on some controversial issues around developing a patient preference health status measure [2] and thus suggested using this to develop agreement on a core set of outcomes for RA Clinical Trials.

The first conference had 3 goals:

(1) To attempt to obtain agreement on the minimum number of outcome measures to be included in all RA clinical trials. This was implemented by a preconference questionnaire, presentation of the evidence on their validity, both small group and plenary discussions on their performance in trials and in individual patients, and then by voting using an electronic voting procedure.

(2) To review the range of magnitude of differences judged to be clinically important by experienced clinicians and clinical investigators. This was implemented by a baseline questionnaire and rank ordering of a series of clinical trials and individual patient scenarios, using a nominal group technique.

(3) To review the extent to which experienced clinicians and clinical investigators feel that aggregate measures (indices) are useful in the assessment of trials and individual patients. This was implemented by presentation of the concepts behind a variety of examples of indices, by questionnaire and a scenario ranking exercise incorporating the results of 3 indices.

The term OMERACT was originally established to mean "Outcome Measures in Rheumatoid Arthritis Clinical Trials". Since then the OMERACT initiative has turned into an international informal network, with working groups and gatherings interested in outcome measurement across the spectrum of rheumatology intervention studies. The acronym has therefore been broadened to now stand for 'Outcome Measures in Rheumatology'.

OMERACT has a 5 member Organizing Committee with members from three continents; it has a 15-member Scientific Advisory Committee composed of international opinion leaders from nine countries. More information on OMERACT is available at our website [3], including full proceedings from the recent conferences, and excerpts from earlier proceedings, all of which are freely downloadable.

## What does OMERACT do?

Agreement regarding the use of standardized endpoints in randomized controlled trials and longitudinal observational studies is extremely important. Their use facilitates comparisons of outcomes across studies to provide the best estimates of benefit and safety of therapeutic interventions across differing patient populations. To improve outcome measurement, OMERACT organizes conferences that take place every two years and rotate around the globe. For these conferences, topics of interest are prepared by groups of experts. These topics are then prepared for the conference by literature review and specification of the points for discussion. Most topics are discussed in workshop format, where the aim is to make explicit the areas of agreement and disagreement, and to prioritize the research agenda. In several areas, the group has taken the lead in actually performing the necessary research to bring back to the conference.

Recently, special interest group meetings have emerged alongside OMERACT conferences to speed up the work. When enough data is available, a full module is organized with the intention to come to consensus on guidelines. In addition, OMERACT hosts a discussion group on outcome measures. OMERACT works under the aegis of the International League for Rheumatology (ILAR), the auspices of the World Health Organization (WHO), and is now in the process of a formal affiliation with the Bone and Joint Decade. OMERACT is linked to the Cochrane Collaboration Musculoskeletal Review Group, where the outcomes endorsed by OMERACT are recommended for use in Cochrane Systematic Reviews [4,5].

## How does OMERACT work?

To reach consensus over what should be measured, and how, i.e., what measures are applicable in trials for each clinical indication, OMERACT has developed the following procedure. First, the organizing committee polls experts and opinion leaders to generate interest in the topic at hand. These individuals then form a committee to guide the subsequent process. From the general domains of health status defined by the "D's" (Discomfort, Disability, Dollar Cost, Death), specific domains are formulated for the topic in question. In each domain, measures are collected and tested for their applicability. The domains and the applicable measures form the basis for the consensus guidelines. The process is data-driven and iterative, and has evolved over the past 14 years. Although not needed when OMERACT was small and the same individuals were involved, as OMERACT has grown and new individuals take on the leadership of taskforces, we have found it helps to break down the process into the different stages of Special Interest Groups, Workshops and Modules. One or more of the executive continue to be actively involved in each task force.

Currently, an initiative starts as a Special Interest Group. A small group of experts initiates the research agenda by literature reviews and validation studies. At the conference, in informal discussions, the research agenda is prioritized and tasks are distributed among interested parties. The next step is a Workshop; where studies are presented that help the formulation and selection of the domains. Again, agreement is reached on priorities in research to be performed. The final step is the Module in which evidence (both from literature and from targeted studies) is presented, and final selection of measures can take place. Both in Workshops and in Modules, plenary presentations are complemented by small group sessions where participants express their views and preferences.

These views are brought back to the plenary session, where a final consensus is formulated with the help of interactive voting using electronic touchpads. In Modules, consensus implies agreement on domains or measures; and in Workshops it means the formulation of a research agenda in areas where data-driven decisions cannot be made. The process is iterative, in that guidelines are forever "preliminary" based on the assumption that future data (sometimes a direct result of the research agenda) will serve to refine or modify them. The work needed to justify a module with voting can be fast tracked and achieved within 12 months if there is sufficient existing data on the performance of the instruments measuring the selected attributes. The new staging of starting with SIGs, with criteria for moving to a Workshop, and the additional requirements to warrant a Module, all reflect the expectation that the process can take up to 6 years or more – this is has been the case with outcomes for adverse effects which has been a focus at every OMERACT meeting since the second OMERACT in 1994.

## When is a measure "endorsed by OMERACT"?

A measure is "endorsed" when it passes the OMERACT Filter in its intended setting.

The OMERACT Filter has three component criteria: Truth, Discrimination, and Feasibility. Each component criteria represents a question to be answered of the measure, in each of its intended settings:

1. Truth: is the measure truthful, does it measure what it intends to measure? Is the result unbiased and relevant? This criterion captures the issues of face, content, construct and criterion validity.

2. Discrimination: does the measure discriminate between situations that are of interest? The situations can be states at one time (for classification or prognosis) or states at different times (to measure change). This criterion captures the issues of reliability and sensitivity to change.

3. Feasibility: can the measure be applied easily, given constraints of time, money, and interpretability? This criterion addresses the pragmatic reality of the use of the measure, one that may be decisive in determining a measure's success.

## What has been achieved?

These 8 OMERACT conferences have succeeded in achieving consensus on core sets of measures for rheumatoid arthritis, osteoarthritis and osteoporosis, psoriasis/psoriatic arthritis [GRAPPA], on psychosocial measures, core set of data for cost-effectiveness evaluations. Taskforces are ongoing in working towards consensus in Surrogate Endpoints in Rheumatology Trials, and Psoriatic Arthritis. Workshops were held on MRI in Ankylosing Spondylitis (AS), Fibromyalgia, Fatigue, Repair in RA, Radiographs/Joint Space Narrowing, Vasculitis, Drug Safety, Scleroderma, Work Productivity, Item Response Theory & Computer Adaptive Testing, Gout, Low Back Pain, Baseline State in RA, Economic Reference Case in AS, Virtual total articular replacement as outcome, Ultrasound, Synovial Tissue, Chemical Biomarkers, MRI in inflammatory arthritis, Single joint response, the Effective Consumer Scale.

The size of the conference has steadily grown from around 80 attendees to 250 attendees at OMERACT 8 – in part due the importance of having leaders in each topic from the Americas, Europe and Australasia and a critical mass of attendees with expertise in the area. Meetings are held intermittently at the World Health Organization to ratify these core sets.

## Impact of OMERACT

Omeract is only one of many initiatives to increase the standardization of outcomes, (e.g. the regular revisions to guidance documents of the regulatory agencies) and sees itself as supporting and facilitating such activities. It is reasonable to argue that OMERACT has provided a key forum for discussions between the different stakeholders, with protected time to focus on this area. Although not quantified, there is little dispute that the number of different primary outcomes has markedly dropped and the OMERACT core sets have been adopted by many other organizations, often being relabeled for their constituency. The Cochrane author guidelines for the Cochrane musculoskeletal Review Group recommend their use.

With few exceptions, the same people have been involved in the development of the American College of Rheumatology [ACR] and European League Against Rheumatism [EULAR]; thus the measures adopted by these organizations are very similar.

The OMERACT process has been emulated by two other independent groups: one working in the field of chronic juvenile arthritis, and one in ankylosing spondylitis. The former group first developed a core set and has continued with response criteria, closely mimicking the process followed by the American College of Rheumatology and OMERACT for adult rheumatoid arthritis. The latter group initially formulated a core set for ankylosing spondylitis, but has now decided to bring their deliberations under the OMERACT umbrella.

Outside of rheumatology, members of the executive committee have lectured on the OMERACT methodology at sessions at the American College of Rheumatology, and various other meetings for experts in community genetics, gastroenterology (non-ulcer dyspepsia), health economics, intensive care medicine, MRI imaging, neurology (neuropathies), and pain measures.

Two meetings were held at the World Health Organization headquarters in Geneva, cosponsored by ILAR. At the first in 1993, the core set agreed upon at OMERACT 1 was ratified. At the second in 2000, the core sets and recommendations arising out of OMERACT 2–4 were ratified.

OMERACT 5 was the first to include a "Young Investigator's Day," thanks to sponsorship of the European Community (Fifth framework). This allowed active young investigators to present recent and ongoing work and be critically appraised by OMERACT faculty. After this day these investigators became full participants in the conference. Funding permitting, we hope to make this a permanent feature of OMERACT conferences.

A small workshop of economics experts was held in New York early in 2001 to lay the groundwork for the module at OMERACT 6. The objective of that meeting was to develop a "reference case" for rheumatology as the first applied version of the generic reference case approach recommended by the US Public Health Service appointed Panel on Cost-Effectiveness in Health and Medicine. This comprises a proposed set of minimum criteria that all economic analyses should include in order to allow comparability across studies in the following categories: model horizon, duration of treatment, extrapolation beyond trial duration, modeling beyond trial duration, synthesis of comparisons where clinical trials do not exist, outcome measures, valuation of health (e.g. QALYs), classification and reporting of adverse events, discontinuation of treatment, therapeutic strategies, population risk stratification, and resource use. This was applied to rheumatoid arthritis at OMERACT 6 and for osteoarthritis and osteoporosis at an NIH funded meeting at the Mayo clinic in April 2003. Funded by EULAR, in 2005 the OMERACT MRI group completed an atlas of MRI images as a special supplement to the Annals of Rheumatic Diseases: The "EULAR-OMERACT rheumatoid arthritis MRI references image atlas". This atlas allows training and further propagation of the reading method endorsed at OMERACT 6.

## The evolution of OMERACT

The governance is evolving to accommodate the increased size of OMERACT, whilst retaining the 'spirit' and special aspects of this informal organization – these include respectful collaboration across all disciplines/sectors, good communication, open decision-making, teamwork, avoidance of duplication, minimal bureaucracy, explicit handling of potential conflicts of interest, continuity, international representation, and wide participation. At OMERACT 8 it was agreed to establish a Steering Group that expands the Executive to include representatives of the leaders of past Omeract Modules/Workshops/SIGs and other stakeholders; for example, lower/middle income countries, major associations, patients, practitioners, private sector. The remit is for this Steering Group to assume overall responsibility for overseeing the development and implementation of policy affecting OMERACT. The Steering Group will have a legal responsibility as the Board of Directors for OMERACT as a registered not-for-profit organisation in Canada. The draft objectives include a) To ensure a systematic mechanism for input from the over-50 individuals who have provided leadership of the many OMERACT groups who have held events at the eight OMERACT meetings so far; b) To provide a forum for discussion of policy with minutes to ensure transparency, whilst retaining the agility of being 'informal'.

Since OMERACT 6, patients have been actively involved in the OMERACT process. The patient group has been mentored by John Kirwan (Bristol, UK), but has developed into an independent 'pressure group' within the OMERACT initiative. At OMERACT 8 they elected their own executive of 6 individuals representing 3 continents, with a Chair who will represent them on the new Steering Group described above. The group publishes a newsletter and has produced an OMERACT glossary for the benefit of patients, but eminently useful for professionals involved in OMERACT as well.

Funding: OMERACT is based on the voluntary contributions of participants – a substantial commitment on probono time is required of the group leading each task force over several years to complete the stages to getting the majority vote on a core set. Support for the conferences has been provided by a mixture of industry, university and governmental support. Fellowship support from trainees has been provided through grants from the European Community and EULAR.

## Conclusion

OMERACT appears to be valued by both the 'producers' and the 'users' of evidence from rigorous clinical trials and observational studies. These efforts to identify, standardize and collect validated outcome measures, as well as definitions of minimal clinically important differences [MCID] in patient reported outcomes, have helped to interpret results from randomized controlled trials to everyday clinical practice. They have also facilitated definitions of clinically meaningful changes, especially in the context of meta-analyses conducted under the Cochrane Collaboration.

Consensus processes conducted within OMERACT facilitate efforts to define and measure improvements in health outcomes across broad populations with musculoskeletal diseases. Linking efforts under the "OMERACT umbrella" with the Bone and Joint Decade initiative will help to identify important unmet needs addressing impairments in physical function and health related quality of life shared by most individuals suffering from chronic inflammatory and/or arthritic conditions.

We should be delighted to compare and contrast this experience in musculoskeletal disorders with other approaches in the same and other fields; however, it does take time – it is important to emphasize that groups wishing to make similar progress in other fields should not expect to achieve consensus or have an impact in the short term

## Competing interests

Peter Brooks would like to declare that he has affiliations with Merck, Genentech, Amgen and Pfizer Pharamceuticals, however he has no competing interests in regards to this manuscript. Vibeke Strand has acted as a consultant to various Pharmaceutical Companies in the past, however she has no specific competing interests in regards to this manuscript.

## Authors' contributions

All authors contributed to the planning of one or more of the OMERACT conferences and to the content of this manuscript.

## Appendix

### OMERACT Proceedings

*Proceedings of the OMERACT Conference on Outcome Measures in Rheumatoid Arthritis Clinical Trials*: *29 April – 3 May 1992; Maastricht*, The Netherlands. *J Rheumatol *1993, 20(3):527–591.

*Proceedings of the OMERACT II Conference on Outcome Measures in Rheumatoid Arthritis Clinical Trials: 30 June – 2 July 1994; Ottawa, Canada. J Rheumatol *1995, 22(5):979–999.

*Proceedings of the OMERACT III Conference on Outcome Measures in Arthritis Clinical Trials: 16 – 19 April 1996; Cairns, Australia. J Rheumatol *1997, 24(4):763–802.

*Proceedings of the OMERACT IV Conference on Outcome Measures in Rheumatology: 16 – 20 April 1998; Cancun, Mexico. J Rheumatol *1999, 26(2):459–507.

*Proceedings of the OMERACT 5 International Consensus Conference on Outcome Measures in Rheumatology 4 – 8 May 2000; Toulouse, France. J Rheumatol *2001, 28(2):394–454.

*Proceedings of the OMERACT 6 International Consensus Conference on Outcome Measures in Rheumatology 11 – 14 April 2002; Gold Coast, Australia*. J Rheumatol 2003, 30(4):865–896.

*Proceedings of the OMERACT 7 International Consensus Conference on Outcome Measures in Rheumatology Clinical Trials; 8 – 12 May 2004; Asilomar, California, USA. J Rheumatol *2005, 32(12):2447–95.

### Related Papers

Østergaard M, Conaghan P: **The EULAR-OMERACT rheumatoid arthritis MRI references image atlas**. *Ann Rheum Dis *2005, 64 Suppl 1: i2–i55.

Saag K: **OMERACT 6 brings new perspectives to rheumatology measurement research**. *J Rheumatol *2003, 30: 639–641.

Brooks P, Hochberg M: **Outcome measures and classification criteria for the rheumatic diseases. A compilation of data from OMERACT (Outcome Measures for Arthritis Clinical Trials), ILAR (International League of Associations for Rheumatology), regional leagues and other groups**. *Rheumatology (Oxford) *2001, 40: 896–906.

Molenaar E, Boers M, Brooks P, Simon L, Strand V, Tugwell P: **Recent developments for optimal endpoints in rheumatoid arthritis clinical studies**. *Dis Management Health Outc *2000, 8: 87–97.

Molenaare E, Van Der Heijde D, Boers M: **Update on outcome assessment in rheumatic disorders**. *Curr Opin Rheumatol *2000; 12:91–8.

Boers M, Brooks P, Strand V, Tugwell P: **The OMERACT Filter for outcome measures in rheumatology. ***J Rheumatol *1998, 25: 198–199.

Boers M, Van Riel PLCM, Felson D, Tugwell P: **Measuring activity of rheumatoid arthritis. ***Baillière's Clin Rheumatol *1995, 9: 305–317.

Boers M, Tugwell P, Felson DT, van Riel PL, Kirwan JR, Edmonds JP, Smolen JS, Khaltaev N, Muirden KD: **World Health Organization and international league of associations for rheumatology core endpoints for symptom modifying antirheumatic drugs in rheumatoid arthritis clinical trials***. J Rheumatol *1994, 21: 86–89.

Tugwell P, Boers M, Baker P, Snider J: **Endpoints in rheumatoid arthritis***. J Rheumatol *1994, 21: 2–8.
